# Expression of NanoLuc Luciferase in *Listeria innocua* for Development of Biofilm Assay

**DOI:** 10.3389/fmicb.2021.636421

**Published:** 2021-02-02

**Authors:** Aleš Berlec, Nika Janež, Meta Sterniša, Anja Klančnik, Jerica Sabotič

**Affiliations:** ^1^Department of Biotechnology, Jožef Stefan Institute, Ljubljana, Slovenia; ^2^Faculty of Pharmacy, University of Ljubljana, Ljubljana, Slovenia; ^3^Biotechnical Faculty, University of Ljubljana, Ljubljana, Slovenia

**Keywords:** *Listeria innocua*, biofilm assay, luciferase, bioluminescence, NanoLuc

## Abstract

Studies of biofilm formation by bacteria are crucial for understanding bacterial resistance and for development of novel antibacterial strategies. We have developed a new bioluminescence biofilm assay for *Listeria innocua*, which is considered a non-pathogenic surrogate for *Listeria monocytogenes. L. innocua* was transformed with a plasmid for inducible expression of NanoLuc luciferase (Nluc). Concentration-dependent bioluminescence signals were obtained over a concentration range of more than three log units. This biofilm assay enables absolute quantification of bacterial cells, with the necessary validation. For biofilm detection and quantification, this “Nluc bioluminescence” method has sensitivity of 1.0 × 10^4^ and 3.0 × 10^4^ colony forming units (CFU)/mL, respectively, with a dynamic range of 1.0 × 10^4^ to 5.0 × 10^7^ CFU/mL. These are accompanied by good precision (coefficient of variation, <8%) and acceptable accuracy (relative error for most samples, <15%). This novel method was applied to assess temporal biofilm formation of *L. innocua* as a function of concentration of inoculant, in comparison with conventional plating and CFU counting, the crystal violet assay, and the resazurin fluorescence assay. Good correlation (*r* = 0.9684) of this Nluc bioluminescence assay was obtained with CFU counting. The limitations of this Nluc bioluminescence assay include genetic engineering of bacteria and relatively high cost, while the advantages include direct detection, absolute cell quantification, broad dynamic range, low time requirement, and high sensitivity. Nluc-based detection of *L. innocua* should therefore be considered as a viable alternative or a complement to existing methods.

## Introduction

Biofilms are the predominant form of bacterial lifestyle that provide bacteria with resistance to adverse environmental conditions, including protection against antimicrobials and disinfectants (de la Fuente-Nunez et al., 2013). To combat the increasing occurrence of resistance to antimicrobials in bacteria, detailed understanding of biofilm formation is crucial. Studies of the processes involved in the different stages of biofilm formation will provide new concepts for development of novel antibacterial strategies.

*Listeria sensu stricto* are ubiquitous Gram-positive bacteria found in soil, water, and vegetation that can colonize mammalian hosts (Haase et al., 2014). Two species, *L. monocytogenes* and *L. ivanovii*, are considered pathogenic, whereas others live as saprophytes only. Infection with *L. monocytogenes* is associated with high hospitalization and mortality rates compared to other foodborne infectious diseases (EFSA and ECDC, 2019). Due to the *L. monocytogenes* pathogenicity and persistence, the use of a non-pathogenic surrogate is preferred for inactivation tests, and especially for in-field studies. *L. innocua* has been proposed as a surrogate organism for *L. monocytogenes*, because they have genetic and physiological similarities, but *L. innocua* lacks the virulence factors of *L. monocytogenes* (Fairchild and Foegeding, 1993; Friedly et al., 2008; Silva-Angulo et al., 2015; Costa et al., 2018) and allows work in Biosafety Level 1 laboratory. *L. monocytogenes* strains differ in their ability to form biofilms (Barbosa et al., 2013; Lee et al., 2019), and an interval of 10^5^ to 10^8^ colony forming units (CFU)/cm^2^ has been reported (Reis-Teixeira et al., 2017). High-biofilm-forming strains produce a dense, three-dimensional biofilm, whereas low-biofilm-forming strains produce a thin, patchy biofilm (Borucki et al., 2003). Increased biofilm formation was observed in serotypes 1/2a and 1/2c (Borucki et al., 2003), or serotypes 3a and 4a (Capita et al., 2019). The biofilm production correlates with environmental conditions, such as temperature and acidic conditions (Borucki et al., 2003; Nilsson et al., 2011; Barbosa et al., 2013). Similar biofilm forming ability was reported for *L. monocytogenes* and *L. innocua* (Costa et al., 2018); however, due to variability between strains this cannot be generalized.

Biofilm assays include direct and indirect methods, whereby the latter require detachment of the microorganisms from the surface prior to their counting (Grudlewska-Buda et al., 2020). Several methods for the evaluation of listerial biofilms have been developed, which include the classical culture-based plate counting method, biomass staining methods (e.g., crystal violet and safranin red), DNA staining methods (e.g., Syto 9), use of chromogenic or fluorogenic metabolic substrates for detection of live bacteria (e.g., tetrazolium salts, resazurin), and also qPCR and digital droplet PCR for quantification of bacterial DNA (Stiefel et al., 2016; Klančnik et al., 2017; Ripolles-Avila et al., 2019; Grudlewska-Buda et al., 2020). New methods for biofilm monitoring are still being introduced; however, both current and new methods have limitations, such as limited accuracy and precision, high detection limits, high cost, long duration, and high workload. Different methods are complementary and assess different pieces of information of the biofilm (e.g., microscopy and plate count) and their outcomes should be merged to obtain a clearer picture of the biofilm. In *Listeria* biofilm research, new methods that enable reproducible detection and quantification of low numbers of bacteria are needed, due to the low biofilm biomass.

Direct detection and quantification of bacteria can be facilitated through genetic engineering and expression of reporter proteins, such as fluorescent proteins and luciferases (Plavec and Berlec, 2019). These enable visualization of the bacteria, and are compatible with fluorescence and bioluminescence colony counting and microplate assays, as well as epifluorescence and confocal microscopy, and flow cytometry. However, the genetic engineering of fluorescent or bioluminescent *Listeria* is not straightforward, and only a few examples have been reported. The pNF8 *Escherichia coli/Listeria* shuttle plasmid that has a strong listerial promoter has been transferred from *E. coli* to *L. monocytogenes* via conjugation to express green fluorescent protein (Fortinea et al., 2000). The same plasmid was transformed into both *L. monocytogenes* and *L. innocua* by optimized electroporation conditions (Ma et al., 2011). A derivative of the pNZ8148 lactococcal plasmid with a strong promoter from lactobacilli was used to express the anaerobic fluorescent protein evoglow-Pp1 in *L. innocua* (Landete et al., 2017).

Fluorescence detection is often used for microscopy; however, it is less appropriate for high-throughput screening due to its lower sensitivity. With low numbers of bacteria, only a weak cumulative fluorescence signal is generated. A stronger signal can be obtained using bioluminescence, whereby the light is produced by luciferase conversion of an appropriate substrate, which provides amplification of the signal. Bioluminescence has already been applied to the monitoring of *Listeria* biofilms and to *in vivo* imaging of *Listeria* infection (Hibma et al., 1996; Hardy et al., 2004; Bron et al., 2006; Riedel et al., 2007). In the most effective example, the Lux operon from *Photorhabdus luminescens* was integrated into the *L. monocytogenes* genome (Bron et al., 2006; Riedel et al., 2007). The downside of this approach was the use of the whole operon (>5,000 bp), and the relatively low dynamic range of 2 log units. Also, none of the reports have provided precise quantification of *Listeria* on the basis of bioluminescence.

Recently, a new luciferase from *Oplophorus gracilirostris* was reported to have superior properties, which is known as NanoLuc (Nluc). Its advantages include its small size (19 kDa), high stability, and high bioluminescence efficiency (150-fold that of other luciferases). Several different applications have already been developed, which include studies of protein–protein interactions, gene regulation, cell signaling, and protein stability (England et al., 2016). However, to the best of our knowledge, the present study is the first example of the use of Nluc for monitoring and quantification of bacterial biofilms.

In the present study, the aim was to produce a non-pathogenic surrogate of *L. monocytogenes*, as *L. innocua* with the expression of Nluc, and to use this surrogate to set up a *L. innocua* quantification assay. This was then applied to monitor biofilm formation in microtiter plates, and included a comparison with other routinely used and established methods for biofilm determination. New information about these biofilms was also obtained. At present, the assay was established in *L. innocua* ŽM39, surrogate for *L. monocytogenes* ŽM58. To test the assay in other strains, recombinant variants will have to be engineered in further studies.

## Materials and Methods

### Bacterial Strains and Culture Conditions

*Listeria innocua* ŽM39 (heat-treated chicken meat; Biosafety Level 1) and *L. monocytogenes* ŽM58 (Klančnik et al., 2015; Biosafety Level 2) were grown at 37°C in tryptic soy broth with shaking, or in the same medium solidified with 1.5% agar in accordance with institutional regulations (ŽM: strain designation in the collection of the Laboratory for Food Microbiology at the Biotechnical Faculty, University of Ljubljana, Slovenia). *E. coli* DH5α was grown at 37°C in lysogeny broth with aeration. Following transformation, erythromycin was added to the growth medium of *L. innocua* (10 μg/mL) and *E. coli* (200 μg/mL) to maintain the selection pressure. Nisin (25 ng/mL) was added to tryptic soy broth to induce Nluc expression in *L. innocua.*

### Molecular Cloning

The Nluc protein (Hall et al., 2012) was back-translated and codon-optimized in the *nluc* gene (GenBank accession number MW139745), which was synthesized as gBlock (Integrated DNA Technologies, Leuven, Belgium; Supplementary Figure S1). Restriction recognition sites were added by PCR amplification, with the primers Nluc-Nco-F (5′-TCCATGGTATTTACCCTTGAAGATTTTG-3′) and Nluc-Xba-R (5′-TTCTAGATTAAGCCAAGATTCTTTCGCATAATC-3′). The PCR amplicon was cloned into the pMSP3545 plasmid (Bryan et al., 2000; Berlec et al., 2006) using the *Nco*I and *Xba*I restriction enzymes (Fast Digest, Thermo Scientific, Vilnius, Lithuania), to provide the plasmid pMSP:Nluc (Supplementary Figure S1), and transformed into *E. coli*. Plasmid DNA was isolated using NucleoSpin Plasmid kit (Macherey-Nagel, Düren, Germany), and sequenced (Eurofins Genomics, Konstanz, Germany). *L. innocua* was transformed by electroporation as previously reported (Ma et al., 2011).

### Measurement of Bioluminescence

The bioluminescence was measured using a plate reader (Infinite M-1000; Tecan, Salzburg, Austria) (settings: luminescence mode; integration time, 1 s; settle time, 150 ms) in white flat-bottomed 96-well plates (Corning, NY, United States). Nano-Glo Luciferase assay reagent (Promega, Madison, United States) was prepared according to the manufacturer instructions, and brought to room temperature. Bacterial samples (50 μL) were mixed with 50 μL reagent, and incubated for 10 min at room temperature before measurement.

### Nluc Bioluminescence Assay

Different concentrations of bacteria were prepared from standardized inocula based on optical density measurements. This range was used for construction of the calibration curve [six serial fivefold dilutions from 5.0 × 10^7^ CFU/mL bacterial dispersion in phosphate-buffered saline (PBS), all in duplicate]. The *L. innocua* concentrations in the samples were determined using linear regression of the calibration curve. The limit of detection (LOD) and limit of quantification (LOQ) were determined as the means of six measurements of the blank increased by 3 standard deviations (LOD) and by 10 standard deviations (LOQ), and calculated using the calibration curve. The accuracy was determined using three known concentrations of *L. innocua* (i.e., 5.0 × 10^6^, 1.0 × 10^6^, 1.0 × 10^5^ CFU/mL), each as two biological and three technical repeats. The relative error was calculated for each measurement. The precision was determined using the same three known concentrations of *L. innocua*, each as six technical repeats. The coefficient of variation was calculated for each measurement. The accuracy and precision were determined twice, on two separate days, to estimate the repeatability of the assay.

### Assays for *L. innocua* Biofilm Formation

Overnight *L. innocua* culture was diluted 1:50 in fresh TSB medium supplemented with erythromycin and nisin. After 8 h of incubation at 37°C with shaking, the bacteria were harvested by centrifugation (10 min at 24°C and 3,000 g) and the OD_600_ was adjusted in fresh PBS to the value of 0.55, corresponding to 5 × 10^7^ CFU/ml (as verified by inoculation on TSA). Different concentrations of *L. innocua* (1.0 × 10^7^, 1.0 × 10^5^, 1.0 × 10^3^ CFU/mL) were obtained by dilution, transferred in 100 μL to white flat-bottomed 96-well plates (Corning, NY, United States) and incubated at 30°C for 4, 8, 24, 48, and 72 h without shaking. At each time, the supernatant was removed and the biofilm was gently washed three times with 100 μL PBS.

For bioluminescence detection, 50 μL PBS was added to the biofilm-containing wells. Freshly prepared bacterial dispersions for the calibration curve were also added to the plates. Then 50 μL Nano-Glo Luciferase assay reagent was added to each well, and the bioluminescence was measured as described above.

For CFU counting, 100 μL PBS was added to the biofilm-containing wells. The plates were parafilm-protected and sonicated for 5 min to detach bound cells. Bacterial suspensions (100 μL) were then transferred to deep-wells containing 900 μL PBS and were serially diluted and plated as previously described (Klančnik et al., 2017). After drying the inoculum, the agar plates were incubated for up to 48 h at 37°C and the grown colonies were counted.

For the crystal violet assay (Kurinčič et al., 2016), the plates were dried after washing at 60°C for 15 min. Crystal violet (0.5%, Merck, Darmstadt. Germany) was added to each well (100 μL) and incubated for 15 min at room temperature. After staining, the plates were washed and dried as described above. The bound crystal violet was dissolved in 100 μL of 33% acetic acid (Merck, Darmstadt. Germany), and the absorbance was measured at 584 nm (VarioskanLux, Thermo Fisher Scientific, Yokohama, Japan).

For the resazurin fluorescence assay for biofilm detection, 100 μL PBS, 10 μL resazurin reagent (prepared from 10 mM resazurin sodium salt; Sigma-Aldrich, Saint Quentin−Fallavier, France), and 0.8 mM menadione (Sigma-Aldrich, Saint Quentin−Fallavier, France) (Kovač et al., 2015) were added, mixed, and incubated for 1 h at 37°C. The fluorescence was measured at 560 nm for excitation and 590 nm for emission (VarioskanLux, Thermo Fisher Scientific, Yokohama, Japan). The minimal reliable signal for the crystal violet and resazurin fluorescence assays were calculated by increasing the background signal by three times the standard deviation of the background (Stiefel et al., 2016).

### Statistical Analyses

Statistical analyses were performed using the GraphPad Prism 5 (GraphPad Software, San Diego, United States). Correlations were determined using Pearson’s coefficients.

## Results

### Heterologous Expression and Functionality of Nluc in *L. innocua*

**FIGURE 1:**
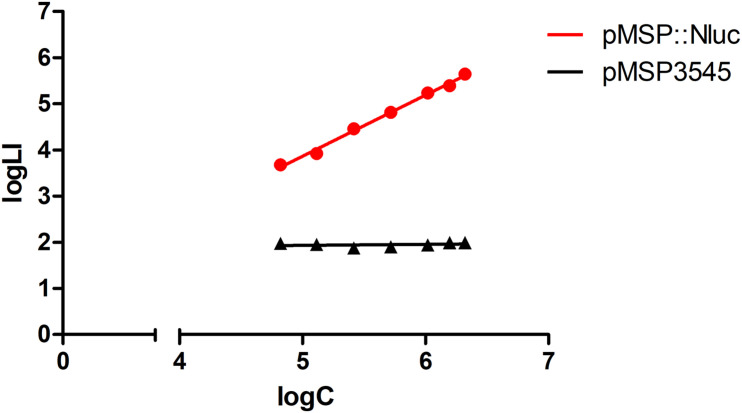
Bioluminescence intensity (logLI) as a function of concentration (logC) of *L. innocua* expressing Nluc (i.e., containing plasmid pMSP:Nluc; red) or control *L. innocua* (i.e., containing empty plasmid pMSP3545; black).

Heterologous expression of Nluc in *L. innocua* was confirmed by comparison of the bioluminescence of *L. innocua* containing the *nluc* gene (plasmid pMSP:Nluc) and the control *L. innocua* containing the empty plasmid. Significantly higher and concentration-dependent bioluminescence intensity was obtained with the Nluc-expressing *L. innocua* (Figure 1), which suggested that this bioluminescence can be used for specific determination of the Nluc-expressing *L. innocua*, and for determination of *L. innocua* concentrations.

### Determination of Nluc Bioluminescence Assay Parameters

An assay for determination of *L. innocua* concentrations was established and validated as previously reported for biological assays (Berlec and Štrukelj, 2014). The parameters determined were linear range interval, limit of detection, limit of quantification, accuracy, and precision, with repeatability determined by performing the assay on multiple days. A linear relationship between the logarithm of bacterial concentration and the logarithm of bioluminescence intensity was established in the *L. innocua* concentration range from 1.6 × 10^4^ to 5.0 × 10^7^ CFU/mL, thereby representing more than 3,000-fold dynamic range in a single assay without the need for dilution of the samples. The calibration curve consisted of six bacterial concentrations obtained with fivefold serial dilutions of the Nluc-expressing *L. innocua* suspension at 5.0 × 10^7^ CFU/mL. The calibration curve was repeatable, with *R*^2^ > 0.99 routinely achieved in separate assays. Two representative examples of the calibration curve and R^2^ are shown in Supplementary Figure S2. The LOD and LOQ were determined on two separate days and are given in Table 1.

**TABLE 1:** Limits of detection (LOD) and quantification (LOQ) of the Nluc bioluminescence assay were calculated, using calibration curves, from the average of six measurements of the blank increased by 3 standard deviations (for LOD) and by 10 standard deviations (for LOQ).

Sampling	LOD (CFU/mL)	LOQ (CFU/mL)
Day 1	1.02 × 10^4^	1.67 × 10^4^
Day 2	1.39 × 10^4^	2.99 × 10^4^

The accuracy of the Nluc bioluminescence assay was determined for three different concentrations of *L. innocua* (i.e., 5.00 × 10^6^, 1.00 × 10^6^, 1.00 × 10^5^ CFU/mL) in two independent biological repeats. Each of the measurements was performed as three technical repeats. To determine the repeatability, the accuracy was determined on two separate days (Table 2). The relative error of the majority of the samples was <15%. Two samples had a relative error from 15 to 30%, and on the second day, the relative errors of the samples with the lowest concentration were over 30% (31.5 and 33.3%).

**TABLE 2:** Accuracy of the Nluc bioluminescence assays on two separate days.

Concentration (CFU/mL)	Determined concentration (CFU/mL)		Relative error (%)	
	Day 1	Day 2	Day 1	Day 2
5.00 × 10^6^	5.33 (± 0.36) × 10^6^	4.25 (± 0.09) × 10^6^	6.7	14.9
5.00 × 10^6^	5.67 (± 0.16) × 10^6^	4.87 (± 0.52) × 10^6^	13.4	2.7
1.00 × 10^6^	1.04 (± 0.01) × 10^6^	7.33 (± 0.17) × 10^5^	4.3	26.7
1.00 × 10^6^	1.04 (± 0.03) × 10^6^	8.60 (± 0.29) × 10^5^	4.1	14.0
1.00 × 10^5^	8.25 (± 0.84) × 10^4^	6.85 (± 0.22) × 10^4^	17.5	31.5
1.00 × 10^5^	8.98 (± 0.70) × 10^4^	6.67 (± 0.22) × 10^4^	10.2	33.3

**Three concentrations were determined, as two biological repeats. Data are means (± standard error).**

To determine the precision, the same three concentrations of *L. innocua* (i.e., 5.00 × 10^6^, 1.00 × 10^6^, 1.00 × 10^5^ CFU/mL) were assayed as six technical repeats. To address repeatability, the precision was also determined on two separate days. The coefficients of variation of all of the samples were well below 8%, which indicated the high precision and good repeatability of this Nluc bioluminescence assay (Table 3).

**TABLE 3:** Precision of the Nluc bioluminescence assays on two separate days.

Concentration (CFU/mL)	Determined concentration (CFU/mL)		Coefficient of variation (%)	
	Day 1	Day 2	Day 1	Day 2
5.00 × 10^6^	4.79 (± 0.27) × 10^6^	4.19 (± 0.01) × 10^6^	5.69	1.99
1.00 × 10^6^	1.02 (± 0.01) × 10^6^	6.78 (± 0.02) × 10^5^	1.27	2.58
1.00 × 10^5^	8.16 (± 0.59) × 10^4^	6.47 (± 0.46) × 10^4^	7.18	7.13

*Data are means (± standard error).*

### Application of the Nluc Bioluminescence Assay for the Monitoring of *L. innocua* Biofilm Formation in Comparison With Other Methods

**FIGURE 2:**
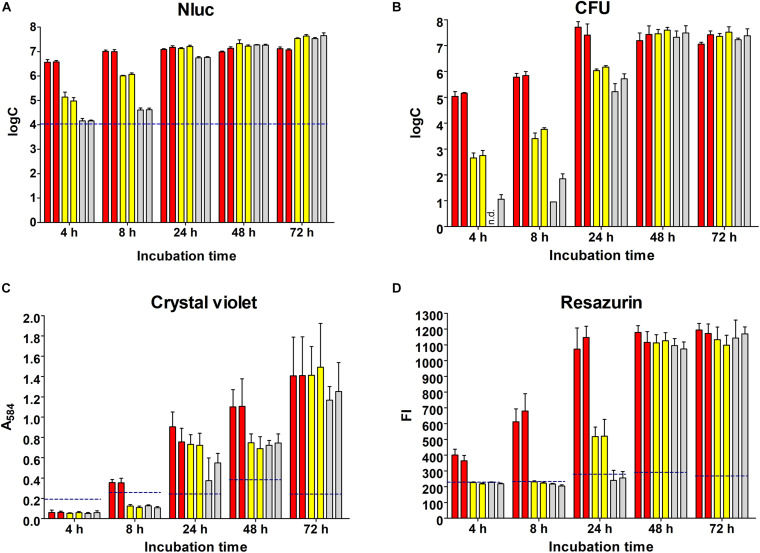
Monitoring of biofilm formation on polystyrene plates over 72 h using the Nluc bioluminescence assay **(A)**, CFU counting assay **(B)**, crystal violet staining **(C)**, and resazurin fluorescence **(D)**. Bars of the same color indicate biological repeats, each performed as three technical repeats. Different concentrations of *L. innocua* were inoculated to trigger biofilm formation (1.0 × 10^7^ CFU/mL, red; 1.0 × 10^5^ CFU/mL, yellow; 1.0 × 10^3^ CFU/mL, gray). Error bars: standard deviation; horizontal dashed blue lines, limit of detection (Nluc) or minimal reliable signal (crystal violet, resazurin). FI, fluorescence intensity; n.d., no CFU on agar plates (i.e., <1 log CFU/mL).

*L. innocua* biofilm formation in polystyrene microtiter plates was initiated with different concentrations of inoculum and monitored over 72 h using the Nluc bioluminescence assay, plating and CFU counting, crystal violet staining, and resazurin fluorescence (Figure 2).

The dynamics of biofilm formation determined with the Nluc bioluminescence assay depended on the concentration of inoculum (Figure 2A). The biofilm formation initiated with the lowest concentration of inoculum required more time to reach the highest concentration of biofilm cells (72 h for 1.0 × 10^3^ CFU/mL inoculum vs. 4 h for 5.0 × 10^7^ CFU/mL inoculant); however, the final concentrations of biofilm cells tended to be higher for biofilms initiated with lower concentrations of inoculum (3.88 × 10^7^ CFU/mL for 1.0 × 10^3^ CFU/mL inoculum vs. 1.25 × 10^7^ CFU/mL for 1.0 × 10^7^ CFU/mL inoculum). The concentrations of the bacterial cells at each time point were calculated from freshly prepared calibration curves, and all of the concentrations determined were > LOD.

**FIGURE 3:**
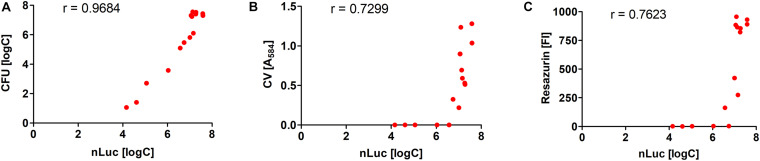
Correlations of *L. innocua* concentrations in biofilms determined with Nluc bioluminescence assay and the CFU counting assay **(A)**, the crystal violet (CV) staining assay **(B)**, and the resazurin fluorescence intensity (FI) **(C)**. The Pearson’s coefficients (*r*) are also shown.

The quantification of the *L. innocua* cells (i.e., CFU) during biofilm formation was also determined with the plate counting method adopting the same experimental set-up (Figure 2B). Similar biofilm formation properties were also observed for control *L. innocua* containing empty plasmid pMSP3545 (Supplementary Figure S3), as well as for *L. monocytogenes* (Supplementary Figure S4). Good correlation for the *L. innocua* concentrations was obtained between the Nluc bioluminescence assay and the CFU counting assay by comparing the same time and the same inoculum concentration (Pearson coefficient *r* = 0.9684; Figure 3A). However, the Nluc bioluminescence assay indicated higher *L. innocua* concentrations by up to 2 log units, particularly during the first 24 h, which might be attributable to the indirect nature of the plate counting method and possibly to the detection of not only live, but also dead and viable-but-non-cultivable cells in the bioluminescence assay, which are not detected with plate counting.

Biofilm formation was also determined using crystal violet, which stains the whole biomass of the biofilm (i.e., bacteria and extracellular matrix). The temporal profile of the biofilm formation here was similar to that obtained with the other methods (Figure 2C). At 4 and 8 h, only one of the measured absorbances was above the minimal reliable signal, which indicated the lower sensitivity of this method in comparison to the Nluc bioluminescence and CFU counting assays. Also, the larger standard errors indicated the lower repeatability of the method.

Temporal biofilm formation determined using the resazurin assay was again similar to that for the other methods (Figure 2D). Similar to crystal violet staining, the resazurin fluorescence assay had lower sensitivity (4, 8, and even 24 h, several values below the minimal reliable signal). This resazurin fluorescence assay provides absolute quantification of bacterial concentration when calibrated against CFU count; however, the assay has a lower dynamic range compared to the Nluc bioluminescence assay when the same concentrations are used for calibration curve, with only 1 log unit increase in the signal (Supplementary Figure S5). The calibration curve was therefore not used for absolute quantification of *L. innocua.*

The correlations between the Nluc bioluminescence *L. innocua* concentrations and crystal violet staining and resazurin fluorescence were lower than for CFU counting (Figure 3). However, these correlations are not directly comparable, as for crystal violet staining and resazurin fluorescence the *L. innocua* concentrations were not determined.

## Discussion

*Listeria* are ubiquitous bacteria, of which *L. monocytogenes* is an important foodborne bacterium that can persist in the environment as biofilms (EFSA and ECDC, 2019). As these are pathogenic bacteria, *L. innocua* has been used as a surrogate in a number of studies (Fairchild and Foegeding, 1993; Friedly et al., 2008; Milillo et al., 2012; Silva-Angulo et al., 2015; Costa et al., 2018). Due to increasing research into bacterial biofilms and the way they are formed and maintained, methods based on microtiter plates are widely used, as these allow large numbers of simultaneous reactions to be carried out. However, the existing methods have some shortcomings, which indicates the need for the development of new methods for such studies. Therefore, this new method based on detection of Nluc bioluminescence of transformed *L. innocua* was developed.

NanoLuc luciferase was effectively expressed in *L. innocua* using the nisin-inducible expression system (Mierau and Kleerebezem, 2005), with a concentration-dependent bioluminescence signal obtained over a broad *L. innocua* concentration range of more than three log units. Validation of the method for quantification of Nluc-expressing *L. innocua* was performed. This method has high sensitivity (1.0 × 10^4^ CFU/mL for detection, 3.0 × 10^4^ CFU/mL for quantification), broad dynamic range (1.0 × 10^4^–5.0 × 10^7^ CFU/mL), good day-to-day repeatability and good precision (coefficient of variation, < 8%). The accuracy is also acceptable (relative error for most samples, <15%), with the exception of the lower concentrations, where the error can be up to 30%.

This Nluc bioluminescence assay was then used to monitor *L. innocua* adhesion and biofilm formation on a polystyrene surface over the course of 72 h. These data were also compared with data obtained with the other commonly used methods for biofilm detection in microtiter plates; i.e., conventional plating and CFU counting, the crystal violet staining assay, and the resazurin fluorescence assay. The kinetics of biofilm formation corresponded to previous studies on *L. monocytogenes* (Chavant et al., 2007; Saá Ibusquiza et al., 2012), which shows comparable biofilm formation to *L. innocua* (Costa et al., 2018), and was also observed in the present study using CFU count. However, to the best of our knowledge, the process of temporal biofilm formation in *L. innocua* has not been described yet. Under the conditions of the present study, the *L. innocua* biofilms formed in 24 h, with cell adhesion already seen in the first 8 h. The trend for biofilm formation over time was comparable between the methods used here. The data for the Nluc bioluminescence assay and plating (CFU counting) correlated well, with higher *L. innocua* concentrations determined using the bioluminescence assay. This can be attributed to the not cultivable cells also detected in the Nluc bioluminescence assay. Conversely, the correlations of the Nluc bioluminescence assay with crystal violet staining and resazurin fluorescence assays were a little lower. In addition to the influence of time, the effect of inoculum concentration on biofilm formation was observed, with particular focus in the first 24 h. At the lower inoculum concentrations, the process of biofilm formation was slower.

Advantages and disadvantages can be seen for all of these assays, which are summarized in Table 4. The methods differ in terms of the necessary research equipment and chemicals, the time needed to perform the assay, the sensitivity and suitability for observing dynamic processes, and the biofilm components that are detected. The Nluc bioluminescence assay detects all phenotypic subpopulations within the growing biofilm, cultivable, viable-but-not cultivable forms and also damaged but not lysed cells. This may be advantageous in evaluation of total biofilm population that includes slowly growing tolerant and persistent cells which are crucial for biofilm persistence (Matereke and Okoh, 2020). Crystal violet stain binds to negative charged molecules in the biofilm biomass and thus detects live and dead cells, as well as the exopolymeric matrix (Costa et al., 2018). The greatest problem with the crystal violet assay is its variability, and therefore deviations in the data, which are due to the operator that performs the assay (Lourenco et al., 2012). This was also evident in the present study, where detectable dye binding was only seen after 24 h. Better reproducibility of these data can be achieved with methods for the determination of live cells. As transition of *Listeria* cells to a viable-but-non-cultivable state is also possible under stress conditions (Highmore et al., 2018), the drawback of the CFU counting assay was that it determines only cultivable live cells. In contrast, all live cells are determined using the resazurin fluorescence assay, which is based on detection of metabolically active cells (Van den Driessche et al., 2014). However, this resazurin method was less sensitive, as at least 10^6^ CFU/mL were required to detect any change in fluorescence. These three methods here are among the most commonly used to determine the formation of bacterial biofilms and for screening for anti-biofilm agents using microtiter plates. Due to the disadvantages and advantages that each of these methods have, the data obtained here suggest the need for application of complementary assays in biofilm studies.

**TABLE 4:** Comparisons of the Nluc bioluminescence assay for biofilm evaluation with the assays using CFU counting, crystal violet staining, and resazurin fluorescence.

Comparison	Assay			
	Nluc	CFU counting	Crystal violet	Resazurin
Mode of detection	Direct	Indirect	Direct	Direct
Biofilm components detected	Live and dead cells^*d*^	Live cells (cultivable)	All biofilm components	Live cells (metabolically active)
Quantification of cells	Absolute	Absolute	Relative	Absolute^*e*^
Dynamic range^*a*^	++	++	+	+
Genetic engineering	Required	Not required	Not required	Not required
Time requirements^*b*^	+	++	+	+
Readout instrument	Plate reader	Not required	Plate reader	Plate reader
Sensitivity^*c*^	++	++	+	+

**^*a*^+, <3 log units; ++, >3 log units. ^*b*^+, <1 h/plate; ++, >1 h/plate. ^*c*^+, <80% of samples here above minimal reliable signal; ++, >80% of samples here above minimal reliable signal. ^*d*^Cultivable and non-cultivable cells containing functional Nluc. ^*e*^Absolute quantification possible (but was not performed here).**

In this study, the Nluc bioluminescence assay enabled temporal monitoring of *L. innocua* biofilm formation in polystyrene plates. Thus, this represents a feasible alternative, or complement, to the existing methods. The limitation of this method includes the requirement for genetic engineering of the bacteria and the relatively high cost, although the cost remains lower than for molecular biology methods (e.g., qPCR), which were not included in this comparison. On the other hand, the Nluc bioluminescence assay has several important advantages over the other methods, which include direct detection mode, absolute cell quantification, broad dynamic range, low time requirement, and high sensitivity. In further studies, the assay will be introduced in other *Listeria* species and strains to confirm its general applicability.

## Data Availability Statement

The datasets presented in this study can be found in online repositories. The names of the repository/repositories and accession number(s) can be found below: https://www.ncbi.nlm.nih.gov/genbank/, MW139745.

## Author Contributions

AB, AK, and JS conceived and designed the research. NJ and MS conducted the experiments. AB and MS analyzed the data. AB wrote the manuscript. All authors read and approved the manuscript.

## Conflict of Interest

The authors declare that the research was conducted in the absence of any commercial or financial relationships that could be construed as a potential conflict of interest.
