# Supplementary material for: Expression of NanoLuc Luciferase in Listeria innocua for Development of Biofilm Assay
Source: Front Microbiol. 2021 Feb 2;12:636421. doi: 10.3389/fmicb.2021.636421 (PMC7901905; doi:10.3389/fmicb.2021.636421)
Supplement: Supplementary file 1 [file Data_Sheet_1.pdf]

Supplementary Material

Expression of NanoLuc luciferase in *Listeria innocua* for development of biofilm assay

Aleš Berlec[1,2]*, Nika Janež[1], Meta Sterniša[3], Anja Klančnik[3], Jerica Sabotič[1]

[1]Department of Biotechnology, Jožef Stefan Institute, Jamova 39, Ljubljana, Slovenia

[2]Faculty of Pharmacy, University of Ljubljana, Aškerčeva 7, Ljubljana, Slovenia

[3]Biotechnical Faculty, University of Ljubljana, Jamnikarjeva 101, Ljubljana, Slovenia

*** Correspondence:**
Corresponding Author
ales.berlec@ijs.si

DOI: 10.3389/fmicb.2021.636421

1 Supplementary Figures

A

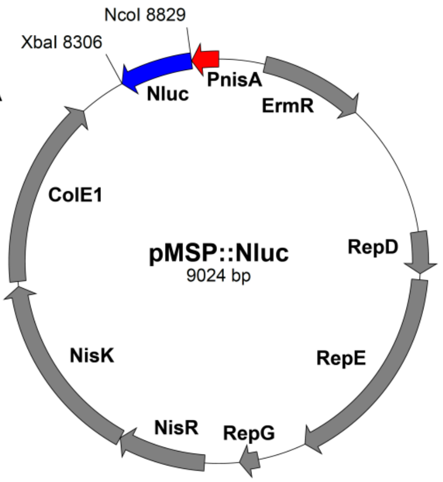

B

```
>Nluc
ATGGTATTTACCCTTGAAGATTTTGTGGGCGATTGGCGTCAAACCG
CCGGATATAATTTGGACCAAGTTTTAGAACAAGGAGGAGTAAGTTC
CTTGTTTCAAAATCTTGGAGTTTCTGTAACACCTATTCAGCGTATT
GTATTGTCAGGTGAAAATGGATTGAAGATAGATATCCACGTCATTA
TTCCTTATGAGGGTCTTTCCGGTGACCAAATGGGACAAATCGAAAA
AATTTTCAAAGTAGTTTATCCTGTTGATGACCATCATTTCAAAGTG
ATTCTACATTATGGTACTCTGGTCATTGATGGCGTTACTCCAAATA
TGATTGACTATTTTGGACGACCTTATGAAGGCATAGCGGTATTCGA
TGGAAAAAAAATTACTGTTACGGGAACTCTTTGGAACGGTAATAAG
ATTATTGACGAACGCCTGATTAATCCTGATGGTTCGCTTCTTTTTC
GTGTTACTATTAATGGTGTCACTGGTTGGCGATTATGCGAAAGAAT
CTTGGCTTAA
```

Supplementary Figure S1. Schematic of pMSP::Nluc plasmid (A) and nucleotide sequence of *nluc* gene (B).

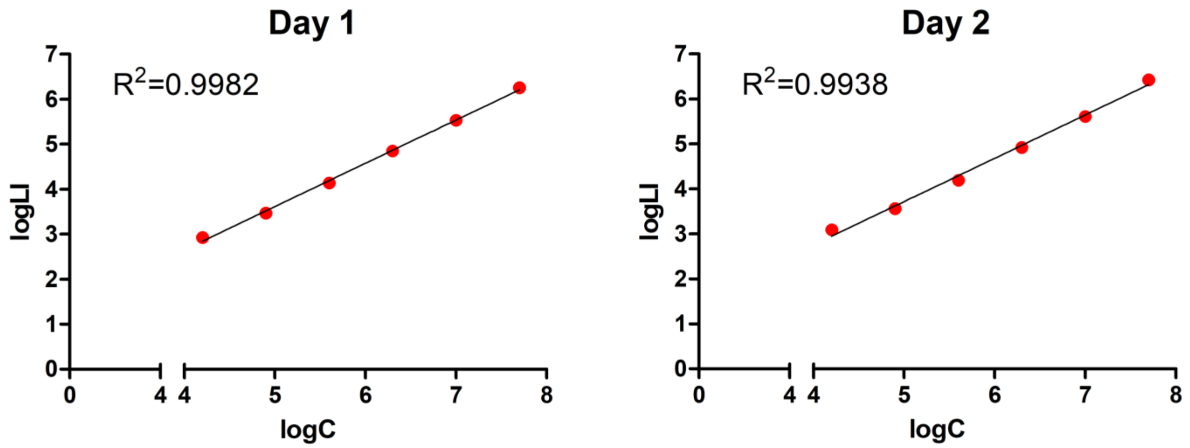

Supplementary Figure S2. Reproducibility of calibration curves for the Nluc bioluminescence assay from two separate days.

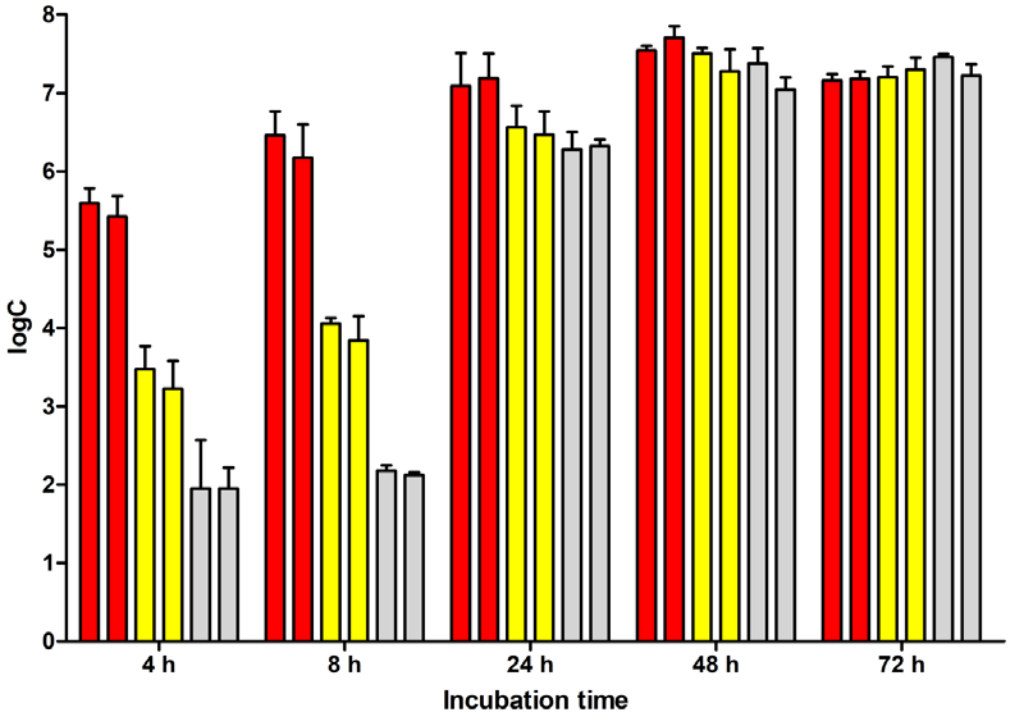

Supplementary Figure S3. Monitoring biofilm formation of control *L. innocua* containing empty plasmid pMSP3545, on polystyrene plates over 72 h using the CFU counting assay. Bars of the same color indicate biological repeats, each performed as three technical repeats. Different concentrations of *L. innocua* were inoculated to trigger biofilm formation (1.0 $\times 10^7$ CFU/mL, red; 1.0 $\times 10^5$ CFU/mL, yellow; 1.0 $\times 10^3$ CFU/mL, gray). Error bars: standard deviation.

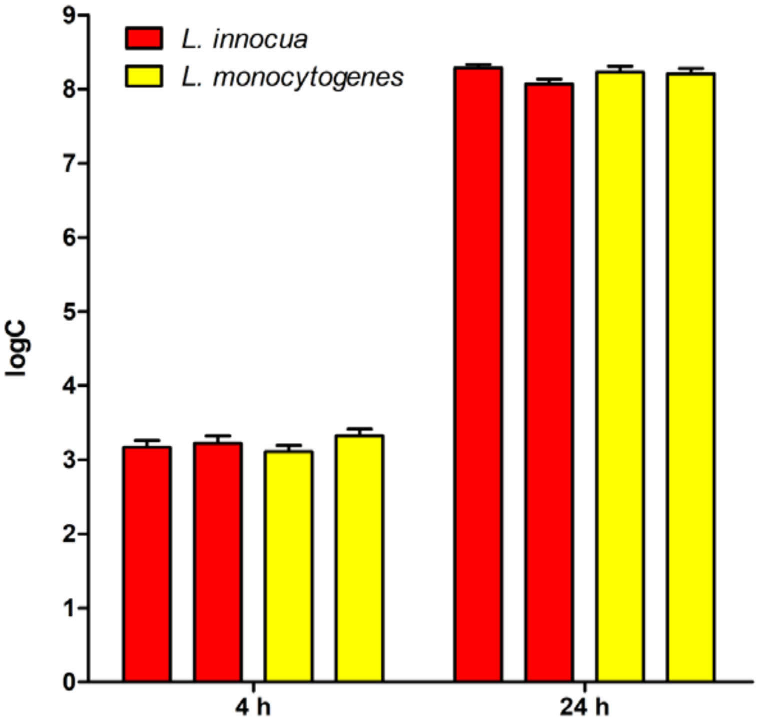

Supplementary Figure S4. Comparison of *L. innocua* (red bars) and *L. monocytogenes* (yellow bars) biofilm formation on polystyrene plates over 24 h using CFU counting. Bars of the same color indicate biological repeats, each performed as six technical repeats. Both species were inoculated at 1.0 $\times 10^5$ CFU/mL. Error bars: standard deviation.

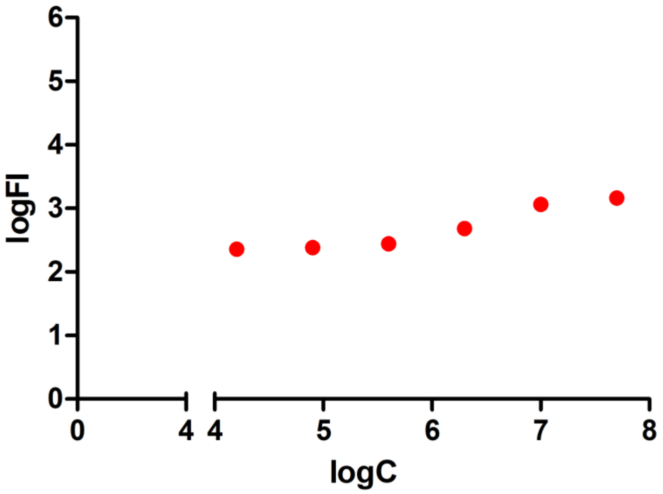

Supplementary Figure S5. *L. innocua* resazurin assay calibration curve based on concentrations applied in NanoLuc luminescence assay. FI: fluorescence intensity.
